# Genetic instability and increased mutational load: which diagnostic tool best direct patients with cancer to immunotherapy?

**DOI:** 10.1186/s12967-017-1119-6

**Published:** 2017-01-21

**Authors:** Giuseppe Palmieri, Maria Colombino, Antonio Cossu, Antonio Marchetti, Gerardo Botti, Paolo A. Ascierto

**Affiliations:** 1 Unit of Cancer Genetics, Institute of Biomolecular Chemistry-National Research Council (ICB-CNR), Traversa La Crucca 3, Baldinca Li Punti, 07100 Sassari, Italy; 2 Institute of Pathology, Azienda Ospedaliero Universitaria (AOU), Sassari, Italy; 3 Center of Predictive Molecular Medicine, Center for Excellence on Ageing and Translational Medicine, University of Chieti-Pescara, Chieti, Italy; 4 Istituto Nazionale Tumori Fondazione Pascale, Naples, Italy

## Abstract

The occurrence of high rates of somatic mutations in cancer is believed to correspond to increased frequency of neo-epitope formation and tumor immunogenicity. Thus, classification of patients with cancer according to degree a somatic hyper-mutational status could be proposed as a predictive biomarker of responsiveness to immunotherapy with immune checkpoint inhibitors. Here, we discuss the suitable and reliable tests easily adoptable in clinical practice to assess somatic mutational status in patients with advanced cancer.

Recently, the load of non-synonymous sequence variants has been significantly associated with clinical benefit from treatment of patients with cancer with immune checkpoint inhibitors In particular, cancer types associated with chronic exposure to external mutagens (i.e. ultraviolet radiations for melanoma or carcinogens and environmental pollutants for lung cancer) or constitutive impairment in genomic integrity (i.e. defective DNA repair mechanisms in a subset of colon cancer) have been reported to preferentially respond to immune checkpoint inhibitors [1–4]. In these conditions, high frequency of mutations seems to determine a higher occurrence of neo-epitope formation and, thus, tumor immunogenicity [5]. Therefore, classification of cancer patients according to their somatic mutational status could be being proposed as a predictive biomarker of responsiveness to anti-cytotoxic T-lymphocyte antigen-4 (CTLA-4) [4] and programmed cell death-1 (PD-1) [3] antibodies.

Although qualitative mutation data on somatic cancer samples are still limited, research efforts aim at defining whether the increased load of the non-synonymous sequence variants may follow distinct mutational patterns or rather represent the consequence of the accumulation of mutations in specific pathways [6, 7]. Detection of specific mutations associated with the response to immunotherapy could pave the way to the development of affordable qualitative biomarkers (presence vs. absence) compared to threshold-depending quantitative parameters. Mutation frequency can be accurately analyzed on tumor tissue samples by next-generation sequencing NGS). Unfortunately, this methodology successfully used for research purposes (indeed, they are now commonly taken into account in vast majority of recently-approved clinical trials) remain, too far away from the practicality of clinical use due to the technical difficulties and necessary expertise usually not available in clinical oncology laboratories.

While in the future NGS may cross the threshold of clinical application, what can be done in the meanwhile?

The following pressing question arises: does a reliable and simple diagnostic test exist ready for use in clinical practice for the assessment of a somatic mutational status?

To date, only the selective identification of patients carrying tumors with genomic instability is practically achievable. The occurrence of alterations impairing the mechanisms involved in maintenance of the genome integrity may induce progressive accumulation of genetic DNA errors and provide a selective advantage for cancer cells during malignant evolution. It has been long known that tumors with non-functional DNA mismatch repair (MMR) present with a higher tendency to bear DNA genomic errors and display a pattern of genomic instability [8]. An efficient MMR apparatus is indeed required for accurate DNA replication during cell proliferation, whereas defects result in increased DNA mutation rates. Microsatellite instability (MSI) inferred by detection of ubiquitous somatic variation in length of microsatellite sequences in tumor DNA compared to the corresponding normal DNA [8, 9], is indicative of inactivating alterations in mismatch repair genes in many unrelated tumor types. The highest prevalence of MSI has been reported in colorectal cancer (ranging from 10 to 15% in sporadic and 70 to 90% in hereditary non-polyposis colon carcinomas, but rarely seen in rectal cancers). Among extra-colonic malignancies, MSI has been described in endometrial (accounting for 20–30% of cases), small bowel (15–25%), gastric (10–20%), ovarian (8–12%), gallbladder (5–8%), prostate (3–8%) cancers as well as in melanoma (varying from 2 to 30% in primary tumors and 20% to up to 70% in metastatic lesions) in Western countries [10, 11].

Considering recent results about the efficacy of the PD-1 inhibitors according to the microsatellite status, the response rate in the MMR proficient colorectal cancer (CRC) and non-CRC cohorts was overall 1% (1/79), with a disease control rate of 13% (10/79) [4, 12–15]. Conversely, the MMR deficient CRC and non-CRC cohorts presented response rates of 58% (15/26) and 55% (12/22), respectively, and disease control rates of 88% (23/26) and 77% (17/22) [4, 12–15]. Further studies on immune checkpoint inhibitors, as single agents or in combination, in expanded cohorts of cancer patients evaluated for MSI are ongoing.

Genetic (allelic deletions, as indicated by loss of heterozygosis in tumor DNA, and/or gene mutations) or epigenetic (functional silencing through promoter hyper-methylation) inactivation of both alleles of the MMR genes leads to MSI at somatic level. The MMR system is composed of 6 MMR genes and their encoded proteins (MLH1, MSH2, MSH3, MSH6, MLH3, PMS2), though inactivation of MLH1 and MSH2 account for over 85% of MSI cases [16].

A correlation between presence of MSI and abnormal MMR gene expression has been widely reported [17–19], strongly suggesting that detection of the MMR proteins could represent a surrogate approach for the identification of tumors with genetic instability. Immunohistochemistry is usually conducted for the main MMR gene products, MLH1 and MSH2, failing thus to ensure full coverage of all MSI cases. Combination of microsatellite analysis and immune histochemical staining for MMR gene products better define the so-called mutator phenotype, most prominently associated with increased DNA mutation rates. In our experience, data from immunohistochemistry using both anti-MLH1 and anti-MSH2 antibodies revealed absent protein expression in about two-thirds of the MSI tumors (either colorectal or endometrial carcinomas) [20–24]. As mentioned above, the MSI tumors present a genomic instability at somatic level due to nonfunctional DNA mismatch repair. Overall, concordance between down-regulation of MLH1/MSH2 gene expression and microsatellite instability varies from 68% to more than 80%, with an average of 75% [19, 25, 26]. One could speculate that lack of complete concordance could be due to various factors: (a) the absence of protein expression requires the inactivation of both alleles of the MMR genes, but the occurrence of deleterious mutations altering MMR gene activity may equally affect the functional mechanisms of DNA repair without impairing protein expression; (b) additional genes may be implicated in defects of replication fidelity (c) staining can be heterogeneous throughout tumor samples, and scoring may not be readily reproducible, particularly in the absence of convincing positive internal control. However, the sensitivity for detection of defective MMR is increased when all four MMR proteins are tested [27].

Collating these findings, it becomes evident that MSI might be considered the only reliable marker of replication errors in human cancers and that a well-conducted microsatellite analysis may yield an accurate detection of genetic instability. MSI testing by polymerase chain reaction (PCR) is considered the gold standard allowing the identification of abnormalities even in the setting of non-truncating protein mutations. For this purpose, a recommended reference panel by the National Cancer Institute (Bethesda panel assay) exists and comprises two mononucleotide repeats (*BAT-25* and *BAT-26*) and three dinucleotide repeats (*D5S346*, *D2S123* and *D17S250*) (Table 1) [28]. Although classification also includes the low-frequency MSI group (if only one of five markers shows instability), presence of MSI should be defined by PCR-based detection of at least two unstable (due to deletions or insertions) microsatellite markers in tumor DNA compared to normal DNA. In Fig. 1, representative examples of microsatellite features are shown. In addition to the amplification of the five polymorphic microsatellite loci of the Bethesda panel assay using 5′ fluorescent labeled primers, according to ThermoFisher Scientific (Waltham, MA, USA) guidelines, a second PCR-based fluorescent multiplex assay which may be reliably used in clinical practice to test MSI is actually represented by the MSI Analysis System, Version 1.2 (Promega Corp., Madison, WI, USA), analyzing seven microsatellite markers (mononucletide repeats: *BAT*-*25*, *BAT*-*26*, *NR*-*21*, *NR*-*24*, and *MONO*-*27*; pentanucleotide repeats: *Penta C* and *Penta D*). In both cases, the PCR products are separated by capillary electrophoresis using an automated sequencer (i.e. 3100 or 3500 Series Genetic Analyzers by ThermoFisher Scientific) and the output data analyzed with specific software (i.e. GeneMapper Analysis Software by ThermoFisher Scientific) to determine MSI status. The PCR-based multiplex assay is also relatively inexpensive (less than 50 euros per patient’s classification) as compared to the four-five fold higher costs of developing NGS-based methodologies.

**Table 1:** Sequence repeats at the five marker loci commonly used for PCR-based microsatellite analysis

Marker	Chromosome location	Gene location	Microsatellite repeat unit	Oligonucleotide primers		Amplicon lenght (bp)
*BAT25*	4p12	*cKIT*	Mononucleotide	Forward	TCGCCTCCAAGAATGTAAGT	118–123
				Reverse	TCTGCATTTTAACTATGGCTC	
*BAT26*	2p16.3–p21	*hMSH2*	Mononucleotide	Forward	TGACTACTTTTGACTTCAGCC	109–114
				Reverse	AACCATTCAACATTTTTAACCC	
*D2S123*	2p16	*hMSH2*	Dinucleotide	Forward	AAACAGGATGCCTGCCTTTA	197–227
				Reverse	GGACTTTCCACCTARGGGAC	
*D5S346*	5q21/22	*APC*	Dinucleotide	Forward	ACTCACTCTAGTGATAAATCGGG	96–122
				Reverse	AGCAGATAAGACAGTATTACTAGTT	
*D17S250*	17q11.2–q12	*BRCA1*	Dinucleotide	Forward	GGAAGAATCAAATAGACAAT	151–169
				Reverse	GCTGGCCATATATATATTTAAACC	

*bp* base pairs

**Fig. 1:**
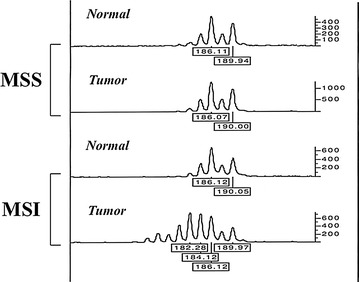
Electropherograms exemplifying microsatellite markers in normal and tumor DNAs. *MSS* microsatellite stability; *MSI* microsatellite instability

While waiting for the application in clinical practice of NGS technology, the standardization of screening approaches based on unique microsatellite panels will improve the classification of genetic instability. This might represent an opportunity to select more homogeneous subsets of unstable patients with a higher mutational load allowing a more accurate assessment of the predictive role of increased mutation rates.

## Abbreviations

CRC: Colorectal cancer
CTLA-4: cytotoxic T-lymphocyte antigen-4
MMR: mismatch repair
MSI: microsatellite instability
MSS: Microsatellite stability
PCR: polymerase chain reaction
PD-1: programmed cell death-1
